# Penicillinase plasmid Australia type in *Neisseria gonorrhoeae* isolated in Poland

**DOI:** 10.1007/s00203-021-02623-w

**Published:** 2022-01-09

**Authors:** Szymon Walter de Walthoffen

**Affiliations:** Department of Medical Microbiology, Medical University of Warsaw, Warsaw, Poland

**Keywords:** *Neisseria gonorrhoeae*, Drug resistance, Beta-lactamase, Penicillinase plasmids

## Abstract

**Purpose:**

*Neisseria gonorrhoeae* is an etiological agent of gonorrhea which remains a major public health problem the mechanisms that determine resistance to drugs of the beta-lactam class, which are recommended for the treatment of gonorrhea, are currently the most important problem in its treatment. Chromosomal mutations are responsible for resistance to ceftriaxone and cefepime. The possibility of mutations in the gene encoding beta-lactamase (*bla*_TEM_) in the penicillinase plasmid may also turn out to be a serious threat.

**Methods:**

The occurrence of resistance encoded on penicillinase plasmid has been investigated. For this purpose, the susceptibility of bacteria was determined and the gene for resistance to beta-lactams as well as the plasmids themselves was typed.

**Results:**

Of the 333 strains tested, 21 (6.3%) had the beta-lactamase gene and produced penicillinase. Two of the beta-lactamase: TEM-1 and TEM-135 occurred among the tested strains of *N. gonorrhoeae*. Most of the known penicillinase plasmid types of *N. gonorrhoeae* were demonstrated: the Asian, the African, the Toronto/Rio plasmids and Australian variants.

**Conclusions:**

In the first 3 years, TEM-1 beta-lactamases dominated in *N. gonorrhoeae*, which were replaced by TEM-135 in the following years of the study. Not all molecular methods are capable of varying the types of penicillinase plasmids. A particularly noteworthy observation is the fact that the Australia-type of penicillinase plasmid (3270 bp) was identified for the first time in Europe, and the second time in the world.

**Electronic supplementary material:**

The online version of this article (10.1007/s00203-021-02623-w) contains supplementary material, which is available to authorized users.

## Introduction

*Neisseria gonorrhoeae* is the etiological agent of gonorrhea which remains a major public health problem. According to the World Health Organization (WHO), there were 87 million cases of gonorrhea worldwide in 2016 (Unemo et al. 2017; Walter de Walthoffen 2021). Gonorrhea often occurs together with other sexually transmitted diseases (Mlynarczyk-Bonikowska et al. 2014; Skulska et al. 2015; Młynarczyk-Bonikowska et al. 2015). Infectious diseases including gonorrhoea are to be covered by an epidemiological surveillance network according to COMMISSION IMPLEMENTING DECISION (EU) 2018/945 of 22 June 2018. *N. gonorrhoeae* has developed multiple resistance mechanisms, which is related to the history of the introduction of new antibiotics into gonorrhoea therapy. Currently, the most significant problem is the production of mechanisms determining resistance to drugs from the group of oxyimino-cephalosporins (Extended spectrum beta-lactamases, ESCs) which are recommended for the treatment of gonorrhea. Chromosomal mutations are responsible for resistance to ceftriaxone and cefepime. Also of great danger is the possibility of a mutation in the penicillinase plasmid, the gene encoding beta-lactamase (*bla*_TEM_) and that mutation can result in the formation of an ESBL-type enzyme (Arlet et al. 1999; Huang and Palzkill 1997; Ohnishi et al. 2010; Muhammad et al. 2014). Currently, gonococcal strains with plasmid- and/or chromosomally mediated resistance to penicillin are common globally (Unemo and Shafer 2014).

Typically, plasmids are irrelevant to their hosts and they only impose an energy burden that can slow cell growth (Kües and Stahl 1989). There is also a correlation between plasmid size and decreased viability at high concentrations of ampicillin to which resistance is caused by plasmids (Diaz Ricci and Hernández 2000; Cheah et al. 1987). Therefore, the occurrence of smaller plasmids (usually in higher copy numbers) in a population of *N. gonorrhoeae* strains may raise a concern about the accelerated evolution of the genes they encode that determine resistance to beta-lactam antibiotics (Walter de Walthoffen 2021).

In penicillinase-producing strains of *N. gonorrhoeae* (PPNG), 7 types of plasmids have been described and they have the *bla*_TEM_ gene in their sequence and they differ in the number of nucleotide base pairs: Asian (7426 base pairs (bp))(Yeung et al. 1986), African (5598 bp) (Alergant et al. 1976; Phillips 1976), Toronto/Rio (5154 bp) (Yeung et al. 1986), and detected in single cases: Nîmes (6798 bp) (Gouby et al. 1986), New Zealand (9309 bp) (Brett 1989), Johannesburg (4865 bp) (Müller et al. 2011), Australia (3269 bp) (Trembizki et al. 2014; Whiley et al. 2014). These plasmids containing the beta-lactamase gene evolved by deletion or insertion of a plasmid fragment. The African and Toronto/Rio-type plasmid was created as a result of various deletions in the Asian plasmid sequence. The Australian plasmid was created as a result of the deletion in the Toronto/Rio plasmid, and the insertion in the Asian plasmid led to the creation of the New Zealand plasmid. The Nîmes-type plasmid was created as a result of the insertion in the African plasmid. These plasmids play an important role in the epidemiology of the spread of PPNG (Pagotto et al. 2000; Lewis 2010) and in the international spread of high-level penicillin resistance (Unemo and Shafer 2011; Berthold 1995).

## Materials and methods

### Bacterial isolates

A total of 333 *Neisseria gonorrhoeae* isolates obtained from the Department of Diagnostics of Sexually Transmitted Diseases, Department of Dermatology and Venereology of the Medical University of Warsaw were tested. These strains were isolated from clinical specimens in the years 2010–2014.

*N. gonorrhoeae* strains were cultured on PolyVitex VCAT3 Chocolate Agar (bioMerieux) and incubated in incubator HCP 105 (Memmert) at 37 °C in a humidified environment with 5% CO_2_ for 24–48 h. *N. gonorrhoeae* was identified using criteria including positive oxidase, acid production from glucose, and Gram-negative staining tests. Confirmed *N. gonorrhoeae* strains were plated on Mueller Hinton Chocolate Agar (MHCA) (Becton Dickinson) and cultured as previously described conditions before the antimicrobial susceptibility test. Isolates were stored at -80° using the Microbank™ system (Pro Lab Diagnostics).

### Antimicrobial susceptibility testing

Benzylpenicillin minimum inhibitory concentrations (MICs) (0.008–64.0 mg/L) for *N. gonorrhoeae* were determined by dilution of the antibiotic method (E-test™) in medium (bioMerieux).

Inoculum of *N. gonorrhoeae* was established by suspending overnight cultures on MHCA medium in 0.9% McFarland's 0.5 standard density saline. The inoculum was inoculated with swabs onto MHCA-containing plates and then E-test™ was placed.

The plates were incubated for 18–24 h at 37 °C in anaerostats, in a gas atmosphere containing 15% O_2_, 75–80% N_2_, 5–10% CO_2_. The Cefinase™ identification test (bioMerieux) was used to detect beta-lactamase-producing *Neisseria gonorrhoeae strains* (PPNG) [29]. The MIC was calculated according to the European Committee for Antimicrobial Susceptibility Testing (EUCAST) guidelines EUCAST "European Committee on Antimicrobial Susceptibility Testing Breakpoint tables for interpretation of MICs and zone diameters Version 8.1, valid from 2018–05–15".

### DNA isolation

DNA was extracted from bacterial suspensions of PPNG strains using a Genomic Mini DNA extraction kit (A&A Biotechnology, Poland). The method is based on the fact that nucleic acids bind to the silica membrane in the presence of chaotropic salts.

### PCR detection of bla_TEM_ gene

The PCR reaction was performed on a C 1000TM ThermalCycler (BIO-RAD, USA). Each 25 µL sample contained 1.5 µL of test DNA, 2.5 µL of 10 × buffer (MBI Fermentas, Lithuania), 2.5 µL of 1.5 mM MgCl_2_ (MBI Fermentas, Lithuania), 0.1 µL of 1.25 U Taq DNA polymerase (MBI Fermentas, Lithuania), 1 µL of 200 µM dNTPs (MBI Fermentas, Lithuania), 0.5 µL of each primer (oligo.pl, Institute of Biochemistry and Biophysics of the Polish Academy of Sciences IBB PAS, Warsaw), 16.4 µL of deionized H_2_O. The following parameters were used in the PCR reaction: 94 °C, 5 min; 35 cycles of 94 °C, 30 s, 57 °C, 30 s, 72 °C, 1 min. The primers with the following sequences were used: F,5′-GTCGCCCTTATTCCCTTTTTTG-3′; R, 5′-TAGTGTATGCGGCGACCGAG-3′ (Nakayama et al. 2012). Electrophoresis in 1% agarose gel (BioRad) was used to visualize the products against size standards (GeneRuler 1 kb DNA Ladder, Fermentas). PCR products stained by ethidium bromide were visualized in a Gel DOC TM XR + Imaging System BIO-RAD instrument.

### Plasmid typing

Plasmid typing was used to determine the polymorphism of mobile genetic elements. The methods used for typing differentiate plasmids by size and on the basis of deletions or insertions in plasmids encoding beta-lactamase in *N. gonorrhoeae* strains. The methods used for typing include whole plasmid amplification, multiplex PCR and plasmid DNA sequencing.

In this study, the method developed by Pagotto et al. (2000) with modification by Ohnishi et al. (2010) and own method were used. The total DNA isolated from the test strains and the following primers *bla*-IR, 5'-TCGTGGTGTCACGCTC GTCG; *bla*-IF; 5'-CTGCAGCAATGGCAACAACGTTG were used for analysis.

Each 50 µL sample contained 3 µL of test DNA, 25 µL of GPB LA DNA polymerase (GenoPlastBiochemicals, Poland)—a kit for amplification of macromolecular DNA fragments, 1 µL of each primer, 20 µL of deionized H_2_O. The following parameters were used in the PCR reaction: 94 °C, 5 min; 10 cycles 94 °C, 20 s, 65 °C, 30 s, 68 °C, 50 s; 20 cycles of 94 °C, 20 s, 65 °C, 30 s, 68 °C, 1 min. The final elongation was carried out at 72 °C, 10 min. To visualize the products, 2% agarose gel electrophoresis was used, in the presence of 1 kb DNA size standards, under the following conditions: 120 V, 400 mA, 240 min, room temperature. After electrophoretic separation, the gel was stained in 150 ml of 1xTBE buffer with 1.5 µl of 10 µg/ml ethidium bromide for 30 min. The stained gel was photographed and documented in the chamber of the transilluminator previously described.

A multiplex PCR method developed by H. Palmer using four primers: BL1 5'-TACTCAATCGGTAATTGGCT-3'; BL2 5'-CACCTATAAATCTCGCAAGC-3'; BL3 5'-CCATAGTGTTGAGTATTGCGAA-3'; BL4 5'-TCATTCGTGCGTTCTAGGA-3' (Palmer et al. 2000) was used for the detection of deletions or insertions in penicillinase plasmids, The total volume of the reaction mixture was 25 µL and contained 1.5 µL of test DNA, 2.5 µL of 10 × buffer (MBI Fermentas, Lithuania), 2.5 µL of 1.5 mM MgCl_2_ (MBI Fermentas, Lithuania), 0.1 µL of 1.25 U Taq DNA polymerase (MBI Fermentas, Lithuania), 1 µL of 200 µM dNTPs (MBI Fermentas, Lithuania), 0.5 µL of each primer, 15.5 µL of deionized H_2_O. The following parameters were used in the PCR reaction: 94 °C, 3 min; 31 cycles of 94 °C, 20 s, 64 °C, 20 s, 72 °C, 1 min. The amplification of the smallest possible products, which allows to detect deletions in the plasmid, is possible by a properly designed elongation phase, and the predicted product size allows detection of a specific plasmid type: BL2 + BL3—958 bp—the Asian plasmid, BL1 + BL3—1191 bp—the African plasmid, BL2 + BL4—650 bp—the Toronto/Rio plasmid. Electrophoresis in 1% agarose gel, 1 kb DNA size standard (Fermentas) was used to visualize the products. PCR products stained with ethidium bromide were visualized in a transilluminator.

Next-Generation Sequencing (NGS) was commissioned and performed for the isolated plasmid DNA of strain NG200 and received resultant was the Sanger method was confirmed in the laboratory of DNA sequencing and Oligonucleotide Synthesis of the Institute of Biochemistry and Biophysics of the Polish Academy of Sciences (IBB PAS). Plasmid DNA was sheared mechanically to appropriate fragments which were used for Paired-End TruSeq libraries construction (KAPA Biosystems, USA) following the manufacturer's instructions. Libraries were sequenced using MiSeq instrument (Illumina, San Diego, CA). The obtained sequence reads were filtered by quality using FastX toolkit (http://hannonlab.cshl.edu/fastx_toolkit) and assembled using Newbler v3.0 software (Roche, USA) resulting in high-quality draft genome assemblies. All of the possible sequence errors and miss assemblies were further manually corrected using Seqman software (DNAStar, USA). Sequencing coverage was 433x.

### Identification of the blaTEM genes

The PCR method developed by V. Speldooren for E. coli beta-lactamase was used to detect the TEM gene (Speldooren et al. 1998). To obtain the complete TEM beta-lactamase sequence (861 bp), three pairs of primers were used for amplification:

*bla*TEM-A 5′-ATAAAATTCTTGAAGAC 7-3′

*bla*TEM-B 5′-AAAACTCTCAAGGATCTT 382-3′

*bla*TEM-C 5′-AAAGATGCTGAAGATCA 301-3′

*bla*TEM-D 5′-TTTGGTATGGCTTCATTC 726-3′

*bla*TEM-E 5′-TTACCAATGCTTAATCA 652-3′

*bla*TEM-F 5′-TTTTTTGCACAACATGGG 1069–3′

The primers were designed to yield three products representing fragments of the entire *bla*_TEM_ gene. The sequences of these products have complementary (common) fragments, allowing the sequenced products to be combined. The flanking primers are compatible with the sequence preceding the beta-lactamase encoding gene *bla*_TEM_-A, and the sequence following *bla*_TEM_-F.

In the PCR reaction, the same proportions of reactants were used for amplification as for penicillin plasmid identification. The following parameters were used in the reaction: 94 °C, 5 min; 36 cycles of 94 °C, 30 s, 42 °C, 1 min, 72 °C, 1 min. The final elongation was 10 min. The resulting PCR products were purified prior to sequencing by the EXO SAP method. The composition of the reaction mixture: 5 µl amplified DNA fragment, 0,5 µl EXO nuclease I, 1 µl Alkaline phosphatase. The purification was carried out in a thermocycler under the following conditions: Incubation I 37 °C for 15 min, Incubation II 85 °C for 15 min.

The purified products were sequenced in the DNA Sequencing and Oligonucleotide Synthesis Laboratory of the Institute of Biochemistry and Biophysics of the Polish Academy of Sciences using the same primers used for the PCR reaction. The sequencing results were analyzed using FinchTV version 1.4.0 and Serial Cloner 1.3.0 which was used to assemble the complete sequence of the *bla*_*TEM*_ gene. The obtained nucleotide sequences of the tested beta-lactamases were compared with the database https://blast.ncbi.nlm.nih.gov.

### Typing of N. gonorrhoeae strains using the NG-MAST method

NG-MAST is a typing method for *N. gonorrhoeae* strains that relies on the analysis of two highly polymorphic genes: *porB* (490 bp) and *tbpB* (390 bp). The following primers were used for amplification:

Por-F5’ 350 CAAGAAGACCTCGGCAA 366 3',

Por-R-5’ 1086 CCGACAACCACTTGGT 1071 3'

TbpB-F-5’ 1098 CGTTGTCGGCAGCGCGAAAAC 1118 3’,

TbpB-F-5′ 1686 TTCATCGGTGCGCTCGCCTTG 1666 3’.

The total volume of the reaction mixture was 50 µL and contained 2 µL of test DNA, 5 µL of 10 × buffer (MBI Fermentas, Lithuania), 5 µL of 1.5 mM MgCl_2_ (MBI Fermentas, Lithuania), 0.2 µL of 1.25 U Taq DNA polymerase (MBI Fermentas, Lithuania), 2 µL of 200 µM dNTPs (MBI Fermentas, Lithuania), 1 µL of each primer, 15.5 µL of deionized H_2_O. The following parameters for the *porB* gene were used in the PCR reaction: 94 °C, 4 min; 25 cycles of 94 °C, 30 s, 58 °C, 30 s, 72 °C, 1 min, and the following parameters were used for the *tbpB* gene: 94 °C, 4 min; 25 cycles 94 °C, 30 s, 69 °C, 1 min, 72 °C, 1 min. To obtain a pure PCR product for sequencing purposes, the purification was performed using the EXO SAP method as discussed above, and the products were sequenced using the same primers used in the PCR reaction at the DNA Sequencing and Oligonucleotide Synthesis Laboratory of the IBB PAS. The sequencing results were analyzed using FinchTV version 1.4.0 software and compared with the database at www.ng-mast.net.

## Results and discussion

Among 333 strains isolated between 2010 and 2014, 21 (6.3%) possessed the beta-lactamase gene and produced penicillinase.

**Fig. 1:**
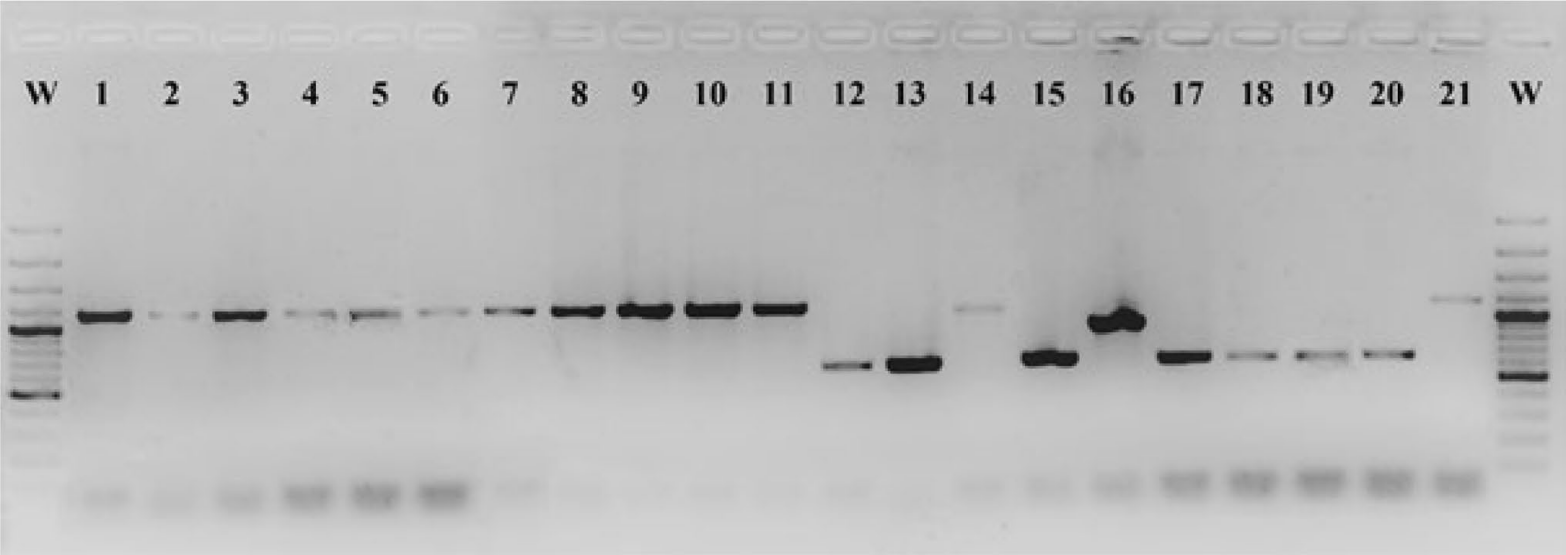
Analysis of plasmids encoding beta-lactamase by amplified plasmid fragment size. W—GeneRuler 1 kb size standard, **1**—Strain number NG1, **2**- Strain number NG2, **3**- Strain number NG6, **4**- Strain number NG13, **5**- Strain number NG24, **6**- Strain number NG28, **7**- Strain number NG32, **8**- Strain number NG58, **9**- Strain number NG112, **10**- Strain number NG113, **11**- Strain number NG119, **14**- Strain number NG171, **21**- Strain number NG328—type Africa (1191 bp), **12**- Strain number NG162, **13**- Strain number NG169, **15**- Strain number NG200, **17**- Strain number NG236, **18**- Strain number NG306, **19**- Strain number NG308, **20**- Strain number NG315—Toronto / Rio type (658 bp), **16**- Strain number NG206—Asia type (958 bp)

The determination of the type of penicillinase plasmid encoding beta-lactamases was performed using a multiplex PCR method detecting deletions in the plasmid. One (5%) of the 21 beta-lactamase-producing strains did not have a deletion in the plasmid and was classified as the Asian type (958 bp) based on the similarity in size of the amplified gene fragment. The deletions in the penicillinase plasmid were detected in 20 strains. The 13 strains (62%) producing beta-lactamase yielded a product size corresponding to the African plasmid (1191 bp), and 7 strains (33%) yielded a product size corresponding to Toronto/Rio plasmid (658 bp) (Fig. 1).

**Fig. 2:**
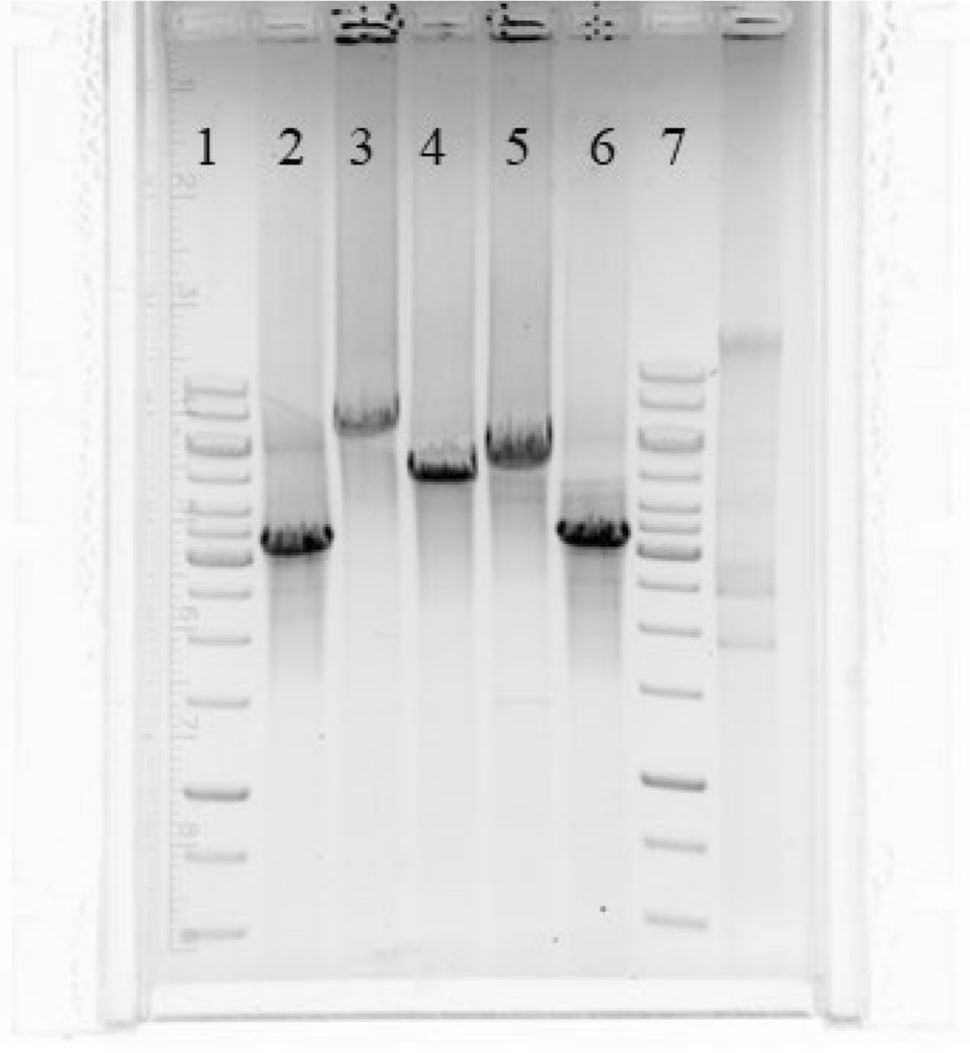
Analysis of beta-lactamase-encoding plasmid types by plasmid size. 1, 7—GeneRuler 1 kb size standard, 2, 6—Strain number NG200—type Australia (a product in size of about 3250 bp), 3—Strain number NG206—Asia type (a product in size of about 7500 bp), 4—Strain number NG162—Toronto/Rio type (a product in size of about 5000 bp), 5—Strain number NG1—type Africa (a product in size of about 5500 bp)

To confirm the above results, the whole plasmid amplification was performed for all 21 beta-lactamase producing strains. The results overlapped for 20 strains. Thirteen strains (62%) had an amplified plasmid size corresponding to the African type (7426 bp), in six strains (28%) the amplified plasmid was classified into the Toronto/Rio type (5154 bp), and one (5%) into the Asian type (742 bp). One NG200 test strain (no.15 in Fig. 1) (5%) had a plasmid size close to the 3500 bp size standard (Fig. 2).

For strain NG200, the deletion detected by multiplex PCR developed by H. Palmer indicated a Toronto/Rio plasmid of 5154 bp (no. 15 in Fig. 1) (Palmer et al. 2000). In contrast, the actual plasmid size obtained by amplification of the entire plasmid was approximately 3500 bp (Fig. 2). NGS was performed for the isolated plasmid DNA of strain NG200 in the Institute of Biochemistry and Biophysics of the Polish Academy of Sciences. By sequencing this strain, three different plasmids of size were identified: 39,071 bp, 3270 bp, 4207 bp, while only the 3270 bp plasmid contained the beta-lactamase coding sequence. The sequence of the plasmid coding for the beta-lactamase is in Appendix A in Supplementary material. The comparison of the obtained sequence in Serial Cloner 1.3.0 and SnapGene programs, allowed to classify the penicillinase plasmid of strain NG200 to the Australian type, based on similarity to the type to Australian GenBank KJ842484. The collective results of the obtained results are presented in Table 1

**Table1:** Comparison of the NG-MAST type with the type of penicillinase plasmid, the type of TEM enzyme and the MIC of penicillin and ceftriaxone

No	Strain number	Data	Type of Plasmid	Type of beta-lactamases	ST NG-MAST	MIC Penicillin mg/L	MIC Ceftriaxone mg/L
1	NG1	2010	Afryka	TEM-1	1478	8	0.004
2	NG2	2010	Afryka	TEM-1	1478	4	0.002
3	NG6	2010	Afryka	TEM-1	1478	32	0.002
4	NG13	2010	Afryka	TEM-1	5421	8	0.002
5	NG24	2010	Afryka	TEM-1	5421	4	0.004
6	NG28	2010	Afryka	TEM-1	5421	4	0.004
7	NG32	2011	Afryka	TEM-1	5421	4	0.002
8	NG58	2011	Afryka	TEM-1	21	8	0.002
9	NG112	2012	Afryka	TEM-1	1478	8	0.008
10	NG113	2012	Afryka	TEM-1	3061	8	0.004
11	NG119	2012	Afryka	TEM-1	21	8	0.002
12	NG162	2013	Toronto/Rio	TEM-135	5624	256	0.004
13	NG169	2013	Toronto/Rio	TEM-135	5624	256	0.004
14	NG171	2013	Afryka	TEM-1	5793	4	0.004
15	NG200	2013	Australia	TEM-135	5624	32	0.004
16	NG206	2013	Azja	TEM-135	12,649	256	0.032
17	NG236	2014	Toronto/Rio	TEM-135	5624	64	0.004
18	NG306	2014	Toronto/Rio	TEM-135	5624	64	0.004
19	NG308	2014	Toronto/Rio	TEM-135	5624	256	0.004
20	NG315	2014	Toronto/Rio	TEM-135	5624	16	0.004
21	NG328	2014	Afryka	TEM-1	16,014	8	0.004

The complete plasmid sequence of strain NG200 [Genbank number (appendix)], obtained during differentiation of the atypical Australian plasmid, showed mutations relative to the available sequence of the originally identified Australian plasmid: GenBank KJ842484 (Trembizki et al. 2014). The Australian plasmid variant (3269 bp) was detected in the PPNG strain studied. There is an adenine insertion at position 1806 in the sequence of the NG200 strain tested relative to the GenBank sequence: KJ842484. This mutation is located in the sequence encoding the replication initiator protein of the RepB family plasmid (GenBank: ARC00143.1) which also has topoisomerase I activity. The Rep 3 region of the RepB protein is responsible for initiating protein replication, whereas the RAMP I III region is responsible for the production of RAMP proteins (Repeat Associated Mysterious Proteins) which are involved in the regulation of the flow of genetic information CRISPR/ssCas (Clustered Regularly-Interspaced Short Palindromic Repeats) by interference (silencing) of genes (Wang and Li 2012).

Using two different electrophoresis-based PCR methods, differences in detected plasmid types were observed. In the multiplex PCR method detecting deletions in the plasmid, the penicillinase plasmid of strain NG200 was classified as the Toronto/Rio type, while using the whole penicillinase plasmid amplification method this result was not confirmed. Only sequencing of the plasmid confirmed that it was an Australian-type variant. The electrophoresis-based PCR techniques mentioned above do not accurately differentiate between *N. gonorrhoeae* plasmid types beyond the most commonly detected types: the Asian, the African, the Toronto/Rio types. Jo-AnneDillon et al., as authors of one of these methods themselves, noted that other types of penicillinase plasmids can only be identified by close genetic similarity as the Asian, the African or the Toronto/Rio plasmids (Dillon et al. 1999). The simultaneous use of two different methods based on electrophoresis (Ohnishi et al. 2010) confirmed the occurrence of the most common plasmid types and allowed to distinguish them from non-standard plasmid types. The most accurate method for differentiating penicillinase plasmid types appears to be the whole plasmid sequencing.

*N. gonorrhoeae* strains, producing TEM beta-lactamase, isolated in 2010—2012 had lower penicillin MIC values than strains isolated in 2013—2014. Differences in MIC values between years correlated with the type of mutation conditioning the TEM-1/TEM-135 enzyme. The results of the obtained types of TEM beta lactamase enzymes are presented in Table 1. Due to the small sample size (*n* = 21), non-parametric Mann–Whitney *U* tests were used in the statistical analysis, with the *p *value lower than the critical significance level (*p* < 0,05). It was observed that the effect of the type of beta-lactamase produced by the tested strains was significant for penicillin sensitivity with a significance level of *p* = 0.000263. No correlation was observed between beta-lactamase type and sensitivity to ceftriaxone *p* = 0.192600.

Most of the known penicillinase plasmid types of *N. gonorrhoeae* were demonstrated: the Asian, the African, the Toronto/Rio plasmids and the Australian variant that has been detected in Europe for the first time and which so far has only been detected in Australia (Trembizki et al. 2014). The Asian, the African, and the Toronto/Rio plasmid types are common worldwide, including in Europe. In France, in PPNG strains isolated between 2010 and 2012, the most common carrier of the beta-lactamase gene is the African plasmid (157/176, 89.2%) in *N. gonorrhoeae*., the Asian-type (13/176, 7.4%) and the Toronto/Rio (6/176, 3.4%) (Micaëlo et al. 2017). Michelle J. Cole et al. identified in PPNG strains isolated in 2012 in England and Wales the same strains as in France but the Asian plasmid was predominant in the tested strains. The Toronto/Rio plasmid encoding the TEM-135 beta-lactamase was predominant in PPNG strains isolated between 2012 and 2014 in Poland, while no European country reported such predominance (Cole et al. 2015). The African plasmid is predominant worldwide as shown by a study conducted by Ibrahim Muhammad et al. on 139 strains isolated between 2000 and 2011 in 15 European countries (*n* = 40), African countries (*n* = 22), Northern and Southern countries (*n* = 10), Asian countries (*n* = 33) and Western Pacific countries (*n* = 31). The African penicillinase plasmid was present in 67.6% of isolates, the Toronto/Rio plasmid in 18.7% of isolates and the Asian plasmid in 13.7% of isolates. No other types of plasmids encoding TEM beta-lactamase were detected in this study (Muhammad et al. 2014). Global studies confirm the dominance of the African plasmid in Bangladesh where more than 90% of isolates from 1997 to 2006 had this plasmid (Ahmed et al. 2010). In studies on strains isolated between 2006 and 2010 in Brazil, only one plasmid type was detected in PPNG strains – the African plasmid (Uehara et al. 2011). In a study by Ricardo Gianecini et al. from Argentina, the prevalence of PPNG isolates was 16.6% in 2008 and increased to 23.2% in 2012. And the plasmid profile revealed two types of circulating plasmids, with the African plasmid dominating 69% among 143 PPNG strains (Gianecini et al. 2015a, b).

## Conclusion

Differences in MIC values between and in type of penicillinase plasmids years correlated with the type of mutation conditioning the TEM-1/TEM-135 enzyme.

The detection of deletions or insertions in penicillinase plasmids using the multiplex PCR method developed by H. Palmer does not completely differentiate the types of penicillinase plasmids. The best method for obtaining a single result is the whole plasmid sequencing.

The Australian plasmid described in this paper was detected for the first time in Europe and for the second time in the world. Further evolution of gonococci may lead to the development of small plasmids encoding beta-lactamases with an extended substrate spectrum without causing a high energy burden on cells, but it may increase the MIC values for antibiotics.

## Electronic supplementary material

Below is the link to the electronic supplementary material.

Supplementary file1 (PDF 31 kb)

## Data Availability

Most of the data is presented in the publication as well as in the appendix. Data on the susceptibility and sequence of beta-lactamase genes will be provided to the interested parties.
