# Supplementary material for: Penicillinase plasmid Australia type in Neisseria gonorrhoeae isolated in Poland
Source: Arch Microbiol. 2022 Jan 9;204(2):130. doi: 10.1007/s00203-021-02623-w (PMC8743264; doi:10.1007/s00203-021-02623-w)
Supplement: Supplementary file 1 — Supplementary file1 (PDF 31 kb) [file 203_2021_2623_MOESM1_ESM.pdf]

TGTGACTGGTGAGTACTCAACCAAGTCATTCTGAGAATAGTGTATGCGGCGACCGAGTTGCTCTTGCCCGGCG
TCAACACGGGATAATACCGCACCACATAGCAGAACTTTAAAAGTGCTCATCATTGGAAAACGTTCTTCGGGGC
GAAAACTCTCAAGGATCTTACCGCTGTTGAGATCCAGTTCGATGTAACCCACTCGTGCACCCAACTGATCTTCA
GCATCTTTTACTTTCACCAGCGTTTCTGGGTGAGCAAAAACAGGAAGGCAAAATGCCGCAAAAAAGGGAATAA
GGGCGACACGAAAATGTTGAATACTCATACTCTTCCTTTTTCAATATTATTGAAGCATTTACCAGGGTTATTGTC
TCATGAGCGGATACATATTTGAATGTATTTAGAAAAATAAACAAATAGGGGTTCCGCGCACATTTCCCCGAAAA
GTGCCACCTGACGTCTAAGAAACCATTATTATCATGACATTAACCTATAAAAATAAGCGTATCACGAGGCCCTT
TCGTCTTCAAGAATTTTATAAACCGTGGAGCGGGCAATACTGAGCTGATGAGCAATTTCCGTTGCACCAGTGCC
CTTCTGATGAAGCGTCAGCACGACGTTCCTGTCCACGGTACGCCTGCGGCCAAATTTGATTCCTTTCAGCTTTGC
TTCCTGTCGGCCCTCATTCGTGCGTTCTAGGATCCTCCGGCGTTCAGCCTGTGCCACAGCCGACAGGATGGTGA
CCACCATTTGCCCCATATCACCGTCGGTACTGATCCCGTCATCAATGAACCGGACTGCCACGCCCTGAGCGTCA
AATTCCTTTATCAGTTGGATCATATCGGCAGTGTCGCGGCCAAGACGGTCGAGTTTCTTAACCAGAATGACATC
ACCTTCCTCCACCTTCATCCTCAGCAAATCCAGCCCTTCCCGGTCTGTTGAACTGCCGGATGCCTTATCGGTAAA
TATACGGTTTATCGCCGTTCTGGTTGGGGCAATGACTTTGGCGGGTTGTGCCTCAACTAGATATACGGACATTT
ACGGCAATCAAAAAAATTTCCTGCTTGATCATTGTAATGGTGAATAGGTAAATTTTCAACCGCTGGGCAGAAAT
GTTTAGCGGTTGTTTTTATTGCGAACCCTTGCAGAACAAGCAAACAAGTTTACTTGCTCTGTAAGGGTTTTGTA
ATTTTAAATCCTGAAAAAAAGAAAAAATTAGGGCTGAAAGCCCCAAAAAGAGCCTTTAGGCTCTATTTTTTTGT
ATTCTTGTTTGTATTCTTGTTTGTAGGCTCTGGCTCCCCTTGTTACACAAGGCTTGCGAGATTTATAGGTGGACA
AATTTATTGTTATAGGTGGACAAATTTATTGTTATAGGTGGACAAATTTATTGTTATAGTGGTCTTTGTTTTTTAT
TGACCACCATTTAACTGTATGGTTATAATGGGACAATCTTTATTTTATGTCCACCTTGATGATATGACAAATGAT
TTAACAGTCCATAAAGCTAACAATTTTGTACAAGCCAGCTATTCAATGACACTTGATGAAATGCGGATTCTGGC
TTTAACTTTGGGGGTTTTTAATCCTAAAAATCCGTCTAAGCGTGGATTTGATTTCACGGTGGCAGATTTTTGTAA
AAGCTTCCCCGATGTAAATCCTGATATAGCTTACACACAAGTTAGAAATGCCGTTTTAAAAATCTCAAAGCGTT
GGGTTACACTGGTTGATAACGAACACGAATTAACAGAAGTAGCTTTAATTCATAAGCGTTCCTATTTTAAAAAA
GAAGGGCGATTTTATATCGAGTTCCACGATGAGCTAATTCCTTACATTTCTGAATTACACGATAACTACACAAA
ATATAAATTGATCAATATTGGGGCTTTGGGTAGTACGCACGCTATTCGCCTTTATGAATTGTGTTCTCAATATCG
TGATACAGGTTGGCGACAAACAAGCGTAGAAGATATTAAAAGTTGGTTAAGTATCTCTGATAAGTACCCACTG
TTTAAGGATTTTAAAAAGCGAGTATTAACTCCATCCATAAATGAAATTAATGCTAAATCCGATCTGCTTGTTGAT
GTTGAACCTATCAAGCGTGGGCGAACAATCGTAGCATTAAAATTCACGATCAAGAGTAAGAAAAGTGCGGTA
AAAATCGAACAGAAACGCCCTGCATTTCCGCATAAAAACAAGTACGGGAAGTTTGTGAAATTGGATACTCAAA
ATCCCAAAATGAGTAATGCTGAATATGGAAACTATGCGAGAGATTGCCTAAAAATCCTTGAAGATTTTTATTCT
GATCTTGCCGATGTTACAACAGAAGATTTACGGCATTATTGGGTTTTTTTGACTAGCAATGCTAGTTTTAGATCA
AAACTTGGTAAGCGGTCAGATTTTTTAAACGAGTTGCAAAATCGAGGGTATAAGATTGTGAACTGTGAATTAG
TTAAAGTGTGAGAGAAGAACAAGATGAATAGCAATTTTATAACAGAGACAGAAAAATTAAGCCGAGAAAAAG
CGGTTAGAGCGGCTATTGATAATAATCGTTTAGAGGGATTAGAACCAAGCCAAGAGTTTATAGATAGGGGTCT
GACGCTCAGTGGAACGAAAACTCACGTTAAGGGATTTTGGTCATGAGATTATCAAAAAGGATCTTCACCTAGA
TCCTTTTAAATTAAAAATGAAGTTTTAAATCAATCTAAAGTATATATGAGTAAACTTGGTCTGACAGTTACCAAT
GCTTAATCAGTGAGGCACCTATCTCAGCGATCTGTCTATTTCGTTCATCCATAGTTGCCTGACTCCCCGTCGTGT
AGATAACTACGATACGGGAGGGCTTACCATCTGGCCCCAGTGCTGCAATGATACCGCGAGACCCACGCTCACC
GGCTCCAGATTTATCAGCAATAAACCAGCCAGCCGGAAGGGCCGAGCGCAGAAGTGGTCCTGCAACTTTATCC
GCCTCCATCCAGTCTATTAATTGTTGCCGGGAAGCTAGAGTAAGTAGTTCGCCAGTTAATAGTTTGCGCAACGT
TGTTGCCATTGCTGCAGGCGTCGTGGTGTCACGCTCGTCGTTTGGTATGGCTTCATTCAGCTCCGGTTCCCAAC
GATCAAGGCGAGTTACATGATCCCCCATGTTGTGCAAAAAAGCGGTTAGCTCCTTCGGTCCTCCGATCGTTGTC
AGAAGTAAGTTGGCAGCAGTGTTATCACTCATGGTTATGGCAGCACTGCATAATTCTCTTACTGTCATGCCATC
CGTAAGATGCTTTTC
